# Competitive Performance of Transgenic Wheat Resistant to Powdery Mildew

**DOI:** 10.1371/journal.pone.0028091

**Published:** 2011-11-23

**Authors:** Olena Kalinina, Simon L. Zeller, Bernhard Schmid

**Affiliations:** Institute of Evolutionary Biology and Environmental Studies, University of Zurich, Zurich, Switzerland; University of Melbourne, Australia

## Abstract

Genetically modified (GM) plants offer an ideal model system to study the influence of single genes that confer constitutive resistance to pathogens on the ecological behaviour of plants. We used phytometers to study competitive interactions between GM lines of spring wheat *Triticum aestivum* carrying such genes and control lines. We hypothesized that competitive performance of GM lines would be reduced due to enhanced transgene expression under pathogen levels typically encountered in the field. The transgenes *pm3b* from wheat (resistance against powdery mildew *Blumeria graminis*) or *chitinase* and *glucanase* genes from barley (resistance against fungi in general) were introduced with the ubiquitin promoter from maize (*pm3b* and *chitinase* genes) or the actin promoter from rice (*glucanase* gene). Phytometers of 15 transgenic and non-transgenic wheat lines were transplanted as seedlings into plots sown with the same 15 lines as competitive environments and subject to two soil nutrient levels. *Pm3b* lines had reduced mildew incidence compared with control lines. *Chitinase* and *chitinase/glucanase* lines showed the same high resistance to mildew as their control in low-nutrient treatment and slightly lower mildew rates than the control in high-nutrient environment. *Pm3b* lines were weaker competitors than control lines. This resulted in reduced yield and seed number. The *Pm3b* line with the highest transgene expression had 53.2% lower yield than the control whereas the *Pm3b* line which segregated in resistance and had higher mildew rates showed only minor costs under competition. The line expressing both *chitinase* and *glucanase* genes also showed reduced yield and seed number under competition compared with its control. Our results suggest that single transgenes conferring constitutive resistance to pathogens can have ecological costs and can weaken plant competitiveness even in the presence of the pathogen. The magnitude of these costs appears related to the degree of expression of the transgenes.

## Introduction

Advances in biotechnology have allowed the introduction of single genes against fungal pathogens into plants [1], [2]. The resulting transgenic plants offer convenient model systems for ecologists to study the effects of such single pathogen-resistance genes on other phenotypic traits of the plants and open up new horizons for gene × environment interaction studies [3].

Resistance to a pathogen might reduce plant fitness when the pathogen is absent from the environment. Such constitutive resistance is often associated with costs resulting from the allocation of resources to unnecessary defense in a pathogen-free environment, making these resources unavailable for other fitness-relevant processes [4]–[6]. Another type of cost of resistance, addressed less often, are ecological costs which arise when resistance affects the interactions between a plant and its biotic or abiotic environment in a way that reduces plant fitness [6]–[8]. Ecological costs are more difficult to study because they might not be apparent under stable growing conditions indoors or on isolated plants where the range of plant × environment interactions is limited [8]. The few studies which reported ecological costs did not control for a common genetic background of resistant and susceptible plants. Moreover, those studies mostly considered induced and not constitutive resistance and thus might have been biased by side-effects of chemical treatments used for defense induction [9]–[11].

Using genetically modified (GM) cereals as a model system allowed us to control the genetic background of experimental lines and to ensure that the GM lines differed only in one resistance gene from non-GM control lines. With this approach we could avoid the problem that resistant plants might differ in multiple resistance and other genes from control plants, which often hampers interpretation in studies of natural populations or in conventional agricultural crops [3], [12]. Furthermore, promoters used with transgenes are able to enhance gene expression hundredfold and more [13], thus providing a possibility to consider not only the effects of gene presence but also of strong gene expression on resistance and its potential costs.

The desired outcome of introducing genes that confer resistance into plants is that the benefits of resistance may outweigh the potential costs in the presence of the pathogen. Under disease pressure, the introduced trait should lead to better pathogen defense and thus increased fitness of the plants carrying the transgene compared with those lacking it, perhaps allowing transgenic plants or their offspring to become invasive in natural habitats [14], [15]. Because the potential advantage will depend on the presence of the pathogen and, if ecological costs arise, on the characteristics of the environment, the competitiveness of the GM plants must be assessed against appropriate non-GM control plants under disease pressure across a range of environments [16], [17]. This has rarely been done in disease-resistant transgenic plants [18]–[20]. Furthermore, due to the complexity of broad-range competition experiments, most studies have so far only tested a very limited number of competitive interactions.

We used a phytometer approach [21], [22] to assess the competitiveness of six transgenic and nine non-transgenic lines and varieties (henceforth both referred to as “lines”) of wheat. The phytometers of the 15 wheat lines were transplanted as seedlings into plots sown with the same 15 lines as competitive environments and subject to two different soil nutrient levels in a full “mechanistic diallel” setting [23], [24]. Phytometers are individual plants planted into a range of environments. Originally used to measure the quality of different *environments* [21], this approach can also be applied to compare the response of different *plants* (genotypes, lines, species) to environmental conditions [25] and here allowed us to measure a wide range of plant characteristics in a large number of environments while at the same time keeping the required area for the field experiment reasonably manageable.

The spring wheat *Triticum aestivum* L. variety Bobwhite SH 98 26, hence abbreviated Bobwhite, transformed with the wheat *pm3b* gene that confers resistance to powdery mildew *Blumeria graminis* f.sp. *tritici* (DC.) Speer [26], and variety Frisal with introduced fungal resistance genes *chitinase* and *glucanase* from barley [27] were used to study the effects of single pathogen-resistance genes on the competitive ability of GM plants. Since the same lines were used as phytometers and competitive environments (full 15×15 mechanistic diallel), it was possible to assess the effect of every line as a competitive environment on the average performance of every line planted as a phytometer into this environment and to estimate mildew infection and competitiveness of individual phytometers of every line surrounded by plants of the other lines (competitive environment).

Apart from providing information about the effects on plant × environment interactions of single pathogen-resistance genes, the assessment of the competitiveness of transgenic and conventional wheat in crop environments contributes to understanding the potential risks associated with offspring of GM plants potentially occurring and competing with conventional wheat in subsequently sown fields. Furthermore, the potential for enhanced performance of phytometers when grown with another line (“away environment”) instead of its own (“home environment”) would suggest a positive effect of growing wheat in line mixtures, an effect abundantly found in biodiversity experiments [28] and, for example, caused by decreased disease levels at the stand level in mixtures [29], [30], (S. Zeller, O. Kalinina & B. Schmid, unpublished data).

The work presented here is part of a joint project of several research groups called “The Wheat Consortium” within the framework of the Swiss National Research Program 59 “Benefits and risks of the deliberate release of genetically modified plants” (www.NRP59.ch). Other research projects within the Consortium have studied agronomic properties of the wheat lines in common agricultural trials, gene × abiotic environment interactions in the glasshouse and in the field [31], disease resistance and gene expression [32] and the impact of the GM lines on other organisms [33]–[35].

Here we asked the following questions: (1) Do the introduced transgenes improve resistance to mildew and do they affect the performance of the phytometers grown under competition (main effects of transgenes)? (2) How do the nutrient and the competitive environments affect resistance to mildew and phytometer competitive performance (main effects of environments)? (3) Do the differences between transgenic and control lines vary across nutrient and competitive environments (overall transgene × environment interactions)? (4) Do transgenic and control lines behave differently if planted into their own rather than into different lines as competitive environments (home vs. away contrast of transgene × environment interactions)? We found that transgenic constitutive resistance to a fungal pathogen can affect plant × environment interactions and reduce the competitiveness of the GM plants which show strong transgene overexpression.

## Materials and Methods

### Plant Material

We used four transgenic lines derived from the Mexican spring wheat variety Bobwhite and two transgenic lines derived from the Swiss variety Frisal in our experiment. Spring wheat *T. aestivum* is a predominantly self-pollinating species with hexaploid genome and growing season from early spring to late summer (in Switzerland). Bobwhite and Frisal were chosen because these varieties are known for high transformation efficiency and regeneration frequency [36], [37]. Furthermore, they are both susceptible to powdery mildew, yet to different degrees (Bobwhite>Frisal).

The transgenic lines of Bobwhite (*Pm3b*#1–4) and their non-transgenic control sister lines (Sb#1–4) were produced by biolistic transformation in four different transformation events. *Pm3b*#1–3 lines carried a single copy of the transgene *pm3b*, and *Pm3b*#4 line carried one full-length and one non-functional truncated copy [32]. Their non-transgenic sister lines were null-segregants that had undergone the same tissue culture processes and thus had acquired the same potential somaclonal variation as their respective transgenic sisters. Southern blot and PCR analysis showed that Bobwhite and the null-segregants did not carry endogenous copies of the *pm3b* gene or other variants of the *pm3* gene [32]. The *pm3b* gene confers race-specific resistance to powdery mildew and was cloned from the hexaploid wheat landrace Chul [26]. The seeds used in this study were obtained from homozygous GM and control lines that had passed through five generations of sexual reproduction by self-pollination.

The performance in monoculture and the transgene expression of the lines *Pm3b*#1–4 have been described by two companion studies [31], [32]. The constitutive ubiquitin promoter from *Zea mays* L. ensured that the transgene was expressed at a high level: *Pm3b* transcript levels were 11, 55 and 5 times higher in *Pm3b*#1, *Pm3b*#2 and *Pm3b*#3, respectively, compared to the donor landrace Chul according to the results of the field assessment of the three *Pm3b* lines and the landrace Chul in 2009 (at that time line *Pm3b*#4 was not available for comparison) [32]. The expression levels of the *pm3b* gene quantified in the leaf samples from the field in 2008 by reverse transcription, quantitative real-time polymerase chain reaction (RT-qPCR) were similar among *Pm3b*#1, *Pm3b*#3 and *Pm3b*#4 lines, while the line *Pm3b*#2 showed around five times higher expression levels [32]. Partial gene silencing and consequent segregation in resistance were observed in the *Pm3b*#3 line, where some plants showed high resistance and others were susceptible to mildew [32]. In monoculture, some unintended effects such as chlorotic leaves and partial male sterility were observed in line *Pm3b*#2 and in a highly resistant subset of *Pm3b*#3. We hypothesized that these unintended effects were related to a very high transgene expression [32].

The GM lines derived from the variety Frisal expressed either a barley seed *chitinase* gene (line A9 *Chi*) or both a *chitinase* and a *β-1,3-glucanase* gene (line A13 *Chi/Glu*) [37]. Chitinases and glucanases are known for their anti-fungal effect. The expression of these pathogenesis-related genes should result in increased quantitative resistance to mildew [27], [38]. The seeds used for the field experiment were obtained from the sixth generation of transgenic lines A13 *Chi/Glu* and A9 *Chi*. No transgene silencing occurred in these lines (C. Diaz Quijano *et al.*, unpublished data). In the absence of sister lines that had undergone the same tissue culture as the transgenic Frisal lines, we used ordinary non-transgenic Frisal plants as the control line. Here we present the results of the first field experiment carried out with these plants. All GM lines used were produced as model plants for the National Research Program 59 and were not intended for agricultural commercialization.

In addition to the 11 lines already mentioned, four further wheat lines were used: ordinary non-transgenic Bobwhite plants that had not passed through tissue culture and the three commercial non-transgenic Swiss varieties: Casana, Fiorina and Toronit. The latter were used as reference “out-groups” to compare differences caused by the transgenes *within* varieties with differences *between* varieties, and thus to verify whether the characteristics of the GM plants fall within the range of natural variation between conventional varieties of wheat, the criterion of the test of equivalence required by the European Food Safety Authority for risk assessment of GM plants [39].

### Field Experiment

The field experiment took place in 2008 at a research station in Zurich-Reckenholz, Switzerland. The 15 wheat lines were sown in 60 plots of 7×1.08 m each, in a randomized complete block design with four replicate blocks. Each plot represented one of the 15 wheat competitive environments for the phytometers. The two edge subplots of 1×1.08 m in each plot were used for a split-plot treatment, fertilizer application vs. control. Fertilizer was applied twice: when the plants had reached phenological stage 11 on the “Zadoks” scale [40] and again when they had reached stage 39, to one of the two subplots in each plot (two times 3 g N m^−2^ as “Ammonsalpeter 27.5”, Lonza, Visp, Switzerland). The natural field soil provided plants with phosphorous, potassium and magnesium (80, 235 and 234 mg kg^−1^, respectively).

In each 1×1.08 m subplot, 400 wheat seeds were sown in six rows with a distance of 18 cm between the rows using an Oyjord plot drill system (Wintersteiger AG, Ried, Austria). Five seedlings per subplot were randomly chosen and marked shortly after germination for later assessment of mildew incidence in the sown competitive environments. All plots were sprayed with the herbicide cocktail Concert SX (40% Thifensulfurone, 4% Metusulfurone-methyl; Stähler Suisse AG, Zofingen, Switzerland) and Starane super (120 g L^−1^ Bromoxynil, 120 g L^−1^ Ioxynil, 100 g L^−1^ Fluroxypyrmetilheptilester; Omya Agro AG, Safenwil, Switzerland) at the beginning of May. Mildew infection occurred naturally. As a subset of these plots (environments but not the phytometers) had provided the plant material for one of our previous publications [31], it was possible to compare the results of the present phytometer study with the results of the assessments of the plants sown in the plots as competitive environments, at least in those cases where the same lines (*Pm3b* and sister lines) and traits were assessed.

### Phytometers

In February 2008, 3600 individual seeds of the 15 wheat lines (the same lines as used in the field plots) were germinated in a climate-controlled glasshouse (day/night temperature: 21/16°C; additional light: 14 h/10 h day/night period, daily watering by hand) at the Institute of Evolutionary Biology and Environmental Studies, University of Zurich, Switzerland. When the seedlings reached phenological stage 11–12 on the Zadoks scale [40] the temperature in the glasshouse was lowered to 5°C to slow down the growth. In March 2008, when the plants in the field reached the same phenological stage and similar size as the plants in the glasshouse, the seedlings were transplanted from the glasshouse to the field plots and inserted into the test environments described in the previous section. These seedlings, grown under standard conditions in the glasshouse, were used as phytometers to assess their phenotypic response to competitive environments and fertilizer application. In our experiment, the phytometers did not differ in their performance (plant height, number of leaves, phenological stage) among the wheat lines at the stage of transplanting and during early stages (phenological stage 14−15) of growth in the field. This indicates that, if the transplanting influenced the growth of the seedlings, all the phytometers responded to it similarly.

Thirty phytometer seedlings representing the 15 wheat lines were introduced into each 1×1.08 m subplot (Figure S1). Before the phytometers were planted, already-established seedlings of the competitive environment were removed from the rows to free space for five phytometers per row (six rows per subplot). Thus, the ratio phytometers:competitors was 30∶370 in the subplot. The distance between neighbouring phytometer plants in a row was 20 cm. As a result, phytometers of each of the 15 lines occurred in each of the 15 lines as competitive environments. The design was thus a full mechanistic diallel [23], [24]. Each phytometer line was represented twice in each subplot.

### Measurements

We recorded plant height and phenological stage [40] of all phytometer plants 53 days after planting. Plant height was measured from the soil level to the highest point of the plant. The incidence of powdery mildew infection was assessed for phytometers and also for marked plants of the sown competitive environment 80 days after planting when infection reached its maximum. It was measured as a presence/absence of the disease symptoms on individual plants, and then a percentage of plants infected with the pathogen out of all the plants was calculated for each wheat line. After ripening, all phytometers were cut at ground level and separated into vegetative and reproductive parts (spikes). All plant material was dried at 80°C (vegetative parts) and 25°C (reproductive parts) and weighed. We counted the spike number per phytometer plant, threshed the reproductive parts, determined the seed number per plant and obtained the total mass of seeds per plant. Hereafter we refer to the total mass of seeds and the seed number per plant as yield and seed number, respectively. The phytometer data were used to characterize the competitiveness of different wheat lines. For each fertilizer treatment and each trait, we calculated the relative performance values for each phytometer line by dividing the subplot means of the line through the mean value this phytometer line reached in its own competitive environment [23], [41], [42]. This was used as a test for home vs. away effects, corresponding to a main-diagonal contrast within the transgene × environment interaction term [43].

### Data Analysis

Data were analyzed with classical mixed-model analysis of variance (ANOVA) using the statistical software GenStat (VSN International Ldt. 2010). The treatment model consisted of the factorially-crossed phytometer lines and competitive environments (mechanistic diallel) and fertilizer application. The error model consisted of phytometer plants nested within subplots, subplots nested within plots and plots nested within blocks. The terms of the treatment model were tested against the appropriate terms of the error model: competitive environment varied among plots, fertilizer application among subplots and phytometer line within subplots (Figure S2). Residual plots were examined to identify outliers and to check if the assumptions of normality and homoscedasticity were fulfilled. Three hierarchical models were used for the analysis (Figure S2): (1) comparing the groups of GM lines with the groups of control lines (i.e. 4 *Pm3b* lines vs. 4 Sb lines, A9 *Chi* and A13 *Chi/Glu* vs. Frisal), (2) and (3) comparing GM and control lines pairwise (i.e. *Pm3b*#1 vs. Sb#1, A9 *Chi* vs. Frisal, A13 *Chi/Glu* vs. Frisal, etc.). All data were log-transformed to fulfill ANOVA assumptions of normality and homoscedasticity. The binary mildew incidence data were analyzed using multiple logistic regression with mixed-model analysis of deviance [44].

First we analyzed the originally measured variables to identify the differences in phytometer performance and the effects of competitive environment and fertilizer application. Then we analyzed the relative performance values (see previous section) to compare the competitive ability of phytometer lines independently of their different performance in “pure stands” (i.e. phytometer plant in its own competitive environment). For this analysis we calculated the log-ratio of away/home as a dependent variable (see e.g. [45]). For each phytometer line × fertilizer × competitive environment combination and for each trait measured, there were four replicate log-ratios according to the four blocks in the field. However, the relative performance means (in percentage, back-transformed from the log-scale) are presented in text and figures.

In June 2008, 1093 out of 3600 phytometer plants were damaged by vandals. These plants were excluded from the analysis of the traits measured after the damage had happened. ANOVA showed that the damage by vandalism occurred randomly across the phytometer lines and did not interfere with the effects of the factors of interest.

## Results

### Mildew Incidence

#### Main effects of transgenes (phytometer lines)

**Figure 1:**
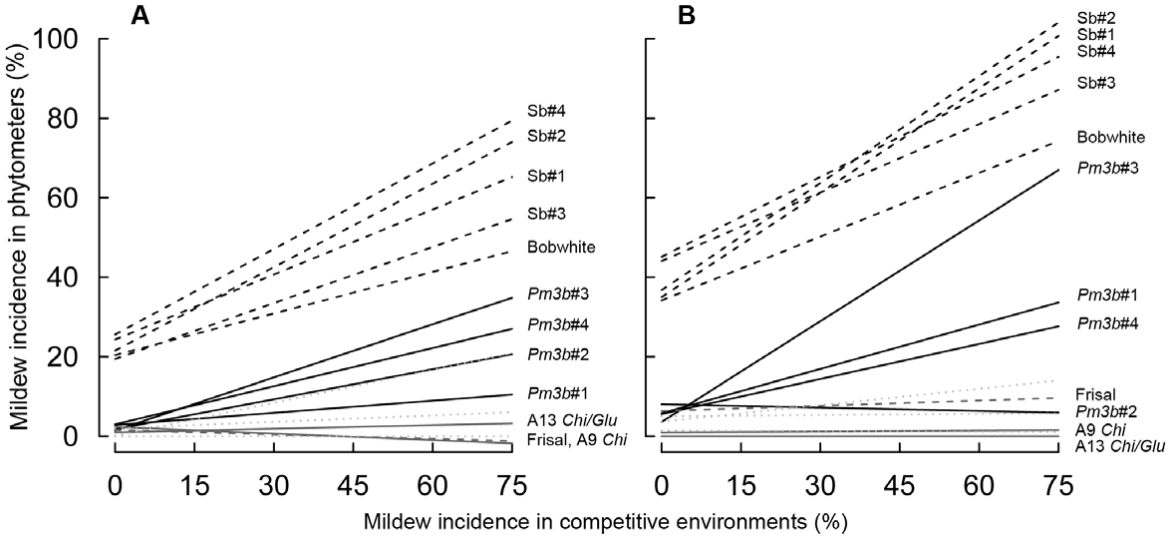
Mildew incidence in phytometers of 15 wheat lines grown with the same lines as competitive environments. Left chart (A): soil with low nutrient level. Right column (B): soil with high nutrient level (fertilized subplots). The mildew incidence in phytometers is plotted as a function of the mildew incidence of the competitive environments (linear regression lines), demonstrating differences among phytometer lines and increased infection in phytometer plants in pathogen-susceptible environments. Mildew incidence is the percentage of plants infected with the pathogen. Solid black lines: lines *Pm3b*#1–4; dashed black lines: lines Sb#1–4 and Bobwhite; solid grey lines: transgenic A9 *Chi* and A13 *Chi/Glu* lines; dashed grey lines: Frisal control line; dotted grey lines: three Swiss conventional wheat varieties (Casana, Toronit and Fiorina).

Powdery mildew incidence reached its maximum in the field 80 days after transplanting of the phytometers. Phytometers carrying the *pm3b* gene showed the desired decrease in mildew incidence (*Pm3b* lines vs. Sb lines phytometer contrast: *P*<0.001); this decrease was up to five-fold compared with control lines (Figure 1). The difference between *Pm3b* lines and Sb lines explained 12.4% of the total variation in mildew incidence and exceeded the variation among the three conventional wheat varieties Toronit, Casana and Fiorina 41.3 times (Table S1).

Each *Pm3b* line had significantly lower mildew incidence than its corresponding Sb line (all pairwise phytometer comparisons of *Pm3b* and Sb lines: *P*<0.001). The four *Pm3b* lines, however, differed significantly from one another in mildew incidence (4 *Pm3b* lines phytometer contrast: *P* = 0.01). Overall, line *Pm3b*#2 had the lowest and line *Pm3b*#3 had the highest mildew scores with, respectively, 6% and 14% of the phytometers infected. The four control Sb lines only marginally differed from each other in mildew incidence (4 Sb lines phytometer contrast: *P = *0.059) and were highly susceptible to the pathogen (up to 62% of the plants infected).

The results obtained with phytometers were similar to the results of other mildew assessments of the same *Pm3b* and Sb wheat lines done in the field at subplot level [31], [32]: the *Pm3b* lines showed higher resistance to the pathogen than the sister lines, *Pm3b*#2 line being the most resistant to powdery mildew among the four GM lines.

The lines derived from the susceptible Mexican variety Bobwhite had a 14-fold increased mildew incidence compared with the lines derived from the Swiss wheat variety Frisal (Bobwhite vs. Frisal phytometer contrast: *P*<0.001).

The lines A9 *Chi* and A13 *Chi/Glu* showed very low mildew incidence (1.5 and 0.7%, respectively), however, mildew incidence was also low in the Frisal control line. Mildew incidence in Frisal plants never exceeded 7% of all phytometers in any line x fertilizer treatment combination (Figure 1). In the fertilized subplots, where the mildew infection was generally higher than in the unfertilized subplots (see next paragraph), the control Frisal line did have higher mildew incidence than the two GM-lines of Frisal (interaction Fertilizer × A9 *Chi* and A13 *Chi/Glu* vs. Frisal: *P* = 0.002; A9 *Chi* and A13 *Chi/Glu* vs. Frisal phytometer contrast: *P* = 0.047 in fertilized environments).

#### Main effects of environments (soil nutrients and wheat competitive environments)

Application of fertilizer led to a twofold increase in mildew incidence of phytometers (main fertilizer effect: *P*<0.001). The competitive environment also affected mildew incidence (main competitive environment effect: *P* = 0.001). Higher mildew rates were observed for the phytometers introduced into mildew-susceptible wheat environments (Figure 1), Sb lines representing the most “infective” environments, in which average mildew incidence among the phytometers reached 29.9%. Mildew occurred 3.6 times more often in phytometers grown in mildew-susceptible Sb environments than in those grown in *Pm3b* plots (*Pm3b* vs. Sb lines competitive environment contrast: *P*<0.001). Mildew incidence did not differ between the phytometers grown in Frisal transgenic and control competitive environments.

#### Overall transgene × environment interactions

The difference in mildew incidence between *Pm3b* and Sb lines increased 1.7-fold with nutrient addition (interaction Fertilizer × *Pm3b* lines vs. Sb lines: *P*<0.001; Frisal results mentioned above). Competitive environment also affected the magnitude of the difference in mildew incidence between *Pm3b* and Sb lines (interaction Competitive environment × *Pm3b* lines vs. Sb lines: *P* = 0.024). This difference was 3.4 times stronger in mildew-susceptible Sb than in mildew-resistant *Pm3b* competitive environments.

### Phytometer Performance

#### Main effects of transgenes (phytometer lines)

**Figure 2:**
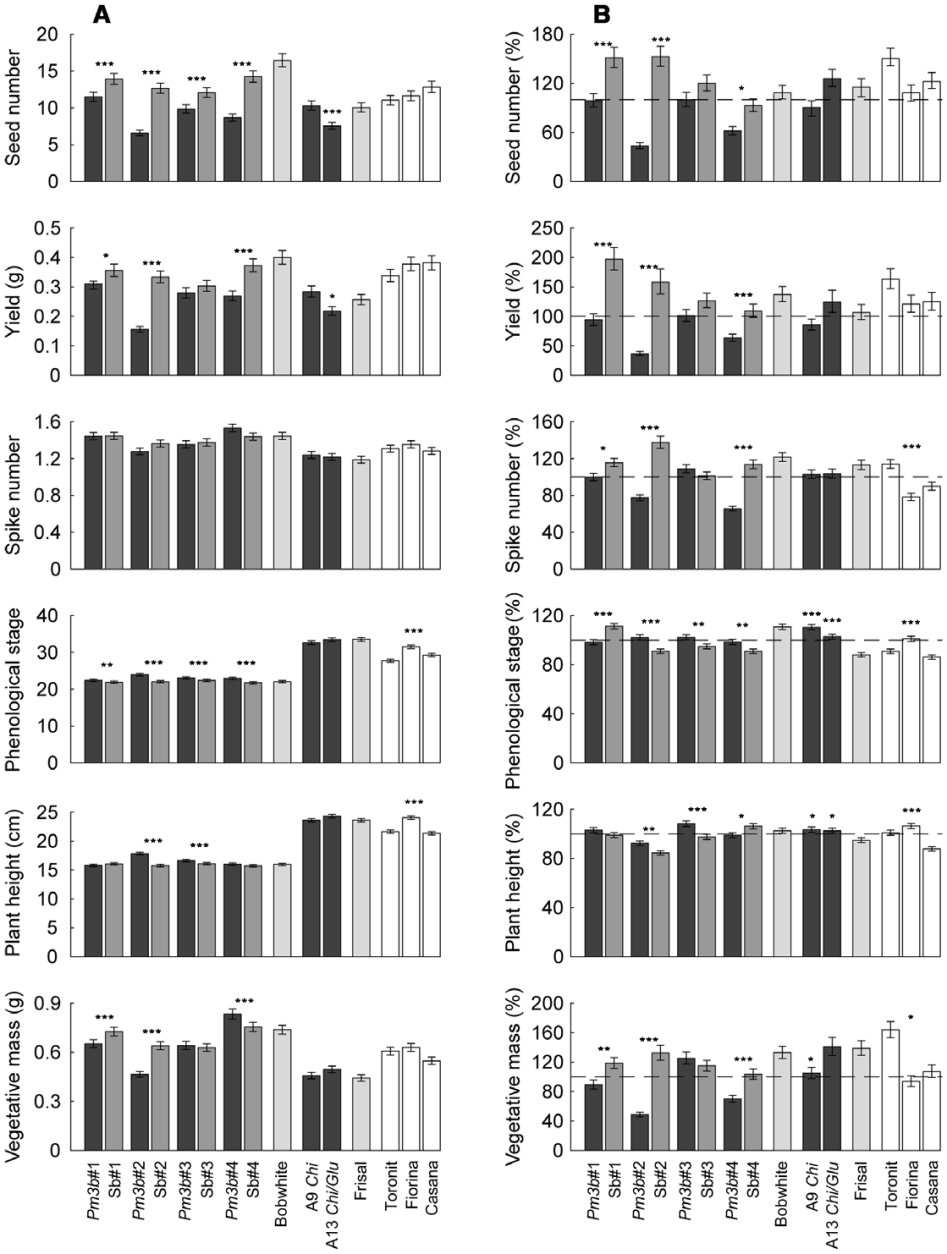
Performance of the 15 wheat lines grown with the same lines as competitive environments. Left column (A): average performance of the transgenic and conventional lines across 15 competitive environments. Right column (B): relative performance of the investigated wheat lines under competition with other lines expressed as a percentage of the estimates in their own environment. The data for high and low nutrient treatments are pooled. Dashed lines denote 100% (i.e. log-ratio = 0: same performance in own and foreign competitive environment). Bars represent means ± standard errors back-transformed from log scale. Five grades of the grey scale indicate groups of wheat lines; from dark to light: transgenic lines, the genetically closest control (sister lines), wheat varieties used for transgene insertion and modern conventional wheat varieties. The significant differences between the *Pm3b* and corresponding control Sb lines, between Frisal and A9 *Chi* line, Frisal and A13 *Chi/Glu* line and among the three conventional varieties Fiorina, Casana and Toronit are shown with asterisks: *** – *P*<0.001, ** – *P*<0.01, * – *P*<0.05.

When planted into competitive environments, transgenic *Pm3b* lines on average developed 45.4% less seeds, 39.4% lower yield and 4.8% lower vegetative mass, and had a more advanced phenological stage and plant height (4.9% and 4.3%, respectively) than control Sb lines (*Pm3b* lines vs. Sb lines phytometer contrasts: *P*<0.001 for all traits) (Figure 2A). In addition, the four *Pm3b* lines differed among each other in performance (4 *Pm3b* lines phytometer contrast: *P*<0.001 for seed number, yield, spike number, vegetative mass and plant height, *P* = 0.023 for phenological stage); the four control Sb lines in contrast differed only in vegetative mass (4 Sb lines phytometer contrast: *P* = 0.018) with lower values observed for the lines Sb#2 and Sb#3 than for the other two lines (Figure 2A; Tables S2 and S3).

The GM line *Pm3b*#2, which exhibited the highest transgene expression [32], had the lowest performance among the four *Pm3b* GM lines: yield was reduced by 53.2%, seed number by 48.1% and vegetative mass by 27.3% compared with the corresponding control Sb#2 (*Pm3b*#2 vs. Sb#2 phytometer contrast: *P*<0.001 for yield, seed number and vegetative mass). This line also had 8.8% more advanced phenological stage and 13.2% taller plants than the control line (*P*<0.001 for phenological stage and plant height). The three other *Pm3b* lines differed less from their controls. The line *Pm3b*#1 had 12.7% reduced yield, 17.5% reduced seed number and 10.2% reduced vegetative mass compared with its sister line Sb#1 (*Pm3b*#1 vs. Sb#1 phytometer contrast: *P* = 0.047, *P* = 0.005 and *P* = 0.015, respectively). *Pm3b*#4 showed 7.9% lower yield and 18.4% lower seed number (*Pm3b*#4 vs. Sb#4 phytometer contrast: *P*<0.001 for yield and seed number) and had slightly advanced phenological stage compared with its sister line Sb#4 (*P* = 0.008 for phenological stage). Line *Pm3b*#3, which had higher mildew incidence than the three other *Pm3b* lines (Figure 1), differed significantly from its sister line Sb#3 only in seed number (*Pm3b*#3 vs. Sb#3 phytometer contrast: *P* = 0.001 for seed number).

The variation explained by the difference between *Pm3b* and Sb lines exceeded the variation among the three conventional wheat varieties Casana, Toronit and Fiorina for the traits yield, seed number and vegetative mass several times (Tables S2 and S3).

The transformed lines derived from the Swiss wheat variety Frisal had an advanced phenological stage and a 1.5-fold increased plant height compared with the lines derived from the Mexican variety Bobwhite. Bobwhite lines, on average, had a 1.3-fold increased seed number per plant and yield, 1.2-fold increased spike number and 1.5-fold increased vegetative mass compared with Frisal lines (Bobwhite vs. Frisal phytometer contrast: *P*<0.001 for all the traits).

Line A9 *Chi* expressing the transgene for chitinase production did not differ in its performance from the Frisal control line, whereas line A13 *Chi/Glu*, expressing two transgenes for chitinase and glucanase, had a 1.2-fold decreased yield and 1.3-fold reduced seed number compared with the Frisal control line (A13 *Chi* vs. Frisal phytometer contrast: *P* = 0.017 for yield, *P*<0.001 for seed number).

#### Main effects of environments (soil nutrients and wheat competitive environments)

Nutrient addition enhanced plant growth and development (main fertilizer effect: *P*<0.001 for all the traits) causing a 2.4-fold increase in yield, 2.3-fold increase in seed number and vegetative mass, 1.4-fold increase in spike number, an advance in phenological stage and a 1.2-fold increase in plant height of the phytometers.

The competitive environment had a strong influence on phytometer growth. Phytometers grown in transgenic *Pm3b* competitive environments had a 1.4-fold yield, 1.5-fold seed number, 1.2-fold spike number and 1.3-fold vegetative mass compared with phytometers grown in Sb competitive environments (*Pm3b* vs. Sb lines competitive environment contrast: *P*<0.001 for vegetative mass, yield and spike number, *P* = 0.001 for seed number). For these traits, the differences between *Pm3b* and Sb competitive environments exceeded the variation among the three conventional-wheat-variety environments (Tables S2 and S3), mirroring the results obtained when analyzing main effects of phytometers (see previous section).

Phytometers which had Frisal lines as competitive environments had delayed phenological development compared with those planted into Bobwhite lines as competitive environments (Bobwhite vs. Frisal competitive environment contrast: *P* = 0.004). The phytometers planted in the different Frisal environments (A9 *Chi,* A13 *Chi/Glu* lines and mother variety) only varied in phenological stage, which was delayed in phytometers grown in the transgenic A9 *Chi* compared with those grown in Frisal environment (A9 *Chi* vs. Frisal competitive environment contrast: *P* = 0.045).

#### Overall transgene × environment interactions

Nutrient addition did not significantly change the magnitude of the differences in performance between *Pm3b* and control Sb lines. However, the plants of the line A9 *Chi* were 4% shorter than those of the Frisal control line in fertilized subplots and did not differ from them in unfertilized subplots (interaction Fertilizer × A9 *Chi* vs. Frisal: *P* = 0.02 for plant height).

The competitive environments Frisal vs. Bobwhite significantly affected the differences in yield, seed number and vegetative mass between *Pm3b* lines and control Sb lines (interaction *Pm3b* lines vs. Sb lines phytometer contrast × Bobwhite vs. Frisal competitive environment contrast: *P* = 0.001 for yield, *P* = 0.002 for seed number, *P* = 0.034 for vegetative mass). The difference between *Pm3b* and Sb phytometers in yield increased 2.1-fold, in seed number 1.7-fold and in vegetative mass 5-fold when the plants were grown in Frisal as compared to Bobwhite environments.

#### Home vs. away contrast of transgene × environment interactions (relative performance)

Overall, yield, seed number and vegetative mass were higher (see term Overall mean in Tables S4 and S5: *P* = 0.009, *P* = 0.032 and *P*<0.001 for log-ratios of yield, seed number and vegetative mass) and plant height and phenological stage were lower in “away” than in “home” environments (*P* = 0.027 for log-ratio of plant height, *P* = 0.001 for log-ratio of phenological stage). This was indicative of higher performance (biomass) due to reduced light competition (lower height) of phytometers in “away” environments.

On average, Bobwhite control lines and conventional Swiss varieties performed better in away than in home environments whereas the opposite was the case for GM lines (Figure 2B): *Pm3b* lines had 52.5% lower relative yield, 44% lower relative seed number, 25.4% lower relative spike number and 32.5% lower relative vegetative mass than the Sb control lines (*Pm3b* lines vs. Sb lines phytometer contrast: *P*<0.001 for log-ratios of yield, seed number, spike number and vegetative mass). However, on average, relative plant height and phenological stage were 4% higher for the *Pm3b* lines than for the Sb lines (*P* = 0.005 and *P* = 0.012, respectively).

Not all lines contributed to the same degree to the mentioned average differences between GM and non-GM lines. The four *Pm3b* lines differed significantly in their relative yield, spike number, seed number, plant height and vegetative mass (4 *Pm3b* lines phytometer contrast: *P*<0.001) (Tables S4 and S5). Lines *Pm3b*#2 and *Pm3b*#4 had the most negative log-ratios for these traits, indicating their weaker performance in competition with the other wheat lines than in “home” environments. In particular, *Pm3b*#2 line had 60% reduced yield, 56.5% reduced seed number, 22.6% reduced spike number and 50% reduced vegetative mass in “away” compared with “home” environments. *Pm3b*#4 line showed 36.5% reduction in yield, 38.3% reduction in seed number, 34.4% reduction in spike number and 29.9% reduced vegetative mass in “away” compared with “home” environments. The performance of the other two *Pm3b* lines in “away” environments was similar to that in their own environment. In particular, line *Pm3b*#3 differed from the Sb#3 control line only by having higher relative phenological stage and plant height (*Pm3b*#3 vs. Sb#3 phytometer contrast: *P* = 0.009 and *P*<0.001 for log-ratios of phenological stage and plant height, respectively). This confirms the results on absolute performance of *Pm3b*#3 line under competition: this line had only minor differences compared with its sister control line.

Frisal transgenic lines A9 *Chi* and A13 *Chi/Glu* had slightly advanced phenological development and plant height in away compared to home environments, whereas the Frisal control line was more phenologically advanced and taller in home than in away environments (A9 *Chi* and A13 *Chi/Clu* vs. Frisal phytometer contrast: *P*<0.001 for log-ratios of plant height and phenological stage). The line A13 *Chi/Glu* and Frisal variety had increased vegetative mass in away as compared to home environments, whereas A9 *Chi* line showed no such effect (A9 *Chi* vs. A13 *Chi/Clu* phytometer contrast: *P* = 0.026; A9 *Chi* vs. Frisal phytometer contrast: *P* = 0.025 for log-ratio of vegetative mass).

Nutrient addition reduced the overall positive away/home log-ratios of yield, seed number, vegetative mass and phenological stage (main fertilizer effect: *P* = 0.014, *P* = 0.001, *P* = 0.007, *P* = 0.021 for log-ratios of yield, seed number, vegetative mass and phenological stage, respectively), indicating that line mixtures may be less beneficial under high than under low soil nutrient conditions.

## Discussion

### Main Effects of Transgenes

Our first question was whether the introduced transgenes improved plant resistance to powdery mildew and whether this resistance incurred any costs for GM plant fitness when the plants were grown under competition and pathogen levels typically encountered in the field. Resistance to mildew was substantially increased in GM lines carrying the *pm3b* transgene, as expected, but generally not in GM lines carrying the *chitinase* and *glucanase* transgenes, presumably because the latter were introduced into the Swiss wheat variety Frisal which already had an elevated level of resistance to the pathogen. The difference in mildew incidence between the GM lines and the control line of Frisal could only be observed in fertilized environments where plants were more susceptible to the pathogen. It is conceivable, therefore, that under higher pathogen pressures the difference between Frisal GM lines and the Frisal control line in pathogen resistance would also have become more apparent.

Increased mildew resistance, however, did not lead to enhanced growth and competitive performance of the tested GM lines in the presence of the pathogen. On the contrary, the plants with *pm3b*-mediated resistance to mildew had on average lower yield and reduced seed number than their corresponding control lines. This suggests that the costs of resistance were high enough to overcome the benefits of being resistant to the pathogen, reducing the plants' fitness and their ability to withstand competition from neighbors. Similar effects, i.e. lower relative fitness under competition, have been previously reported for the plants with chemically induced resistance to pathogens [10], [11]. Analysis of uninfected seedlings [26], [32] had previously shown that the GM lines of Bobwhite expressed the *pm3b* gene constitutively and five- to several-hundred-fold more strongly than did the wheat landrace Chul from which the *pm3b* gene was taken [32]. Although only two *pm* genes have been cloned in wheat [26], [46] and no detailed time-course expression data for indigenous *pm* genes have been published to date, the expression analysis in resistant wheat landrace Chul (S. Brunner *et al*., unpublished data) indicate that the *pm3b* gene is also constitutively expressed with its indigenous promoter. The control Mexican wheat variety Bobwhite and the null-segregants used as sister control lines carried no indigenous *pm* genes. Therefore we suggest that the differences in performance between the *Pm3b* and the control lines could be explained by the high expression of the *pm3b* gene in transgenic lines. However, because we could not compare the performance of our transgenic Bobwhite lines with that of the landrace Chul, we cannot exclude the possibility that even with the original promoter the *pm3b* might have reduced plant performance under the prevailing pathogen pressure.

These costs of resistance also indicate that, at least under the environmental conditions encountered in our field experiment, the mildew-resistant GM lines do not have a higher chance than conventional lines to establish and persist as volunteers in wheat habitats. There is a discussion in the literature if the addition of a single gene can cause a crop to become weedy [47], [48], [49]. Some authors state that weediness arises from many different characters and, therefore, if the species previously had no weedy characteristics, the addition of one or a few genes should not alter its competitiveness to such a large extent as seen in our study [47], [49]. Our results support the contrary point of view that even small genetic changes such as the insertion of a single gene in a new genetic background can cause large ecological alterations affecting genotype × environment interactions [48], [50], [51]. However, in our case the effects of the transgenes were to decrease rather than increase potential weediness in the presence of the pathogen.

**Figure 3:**
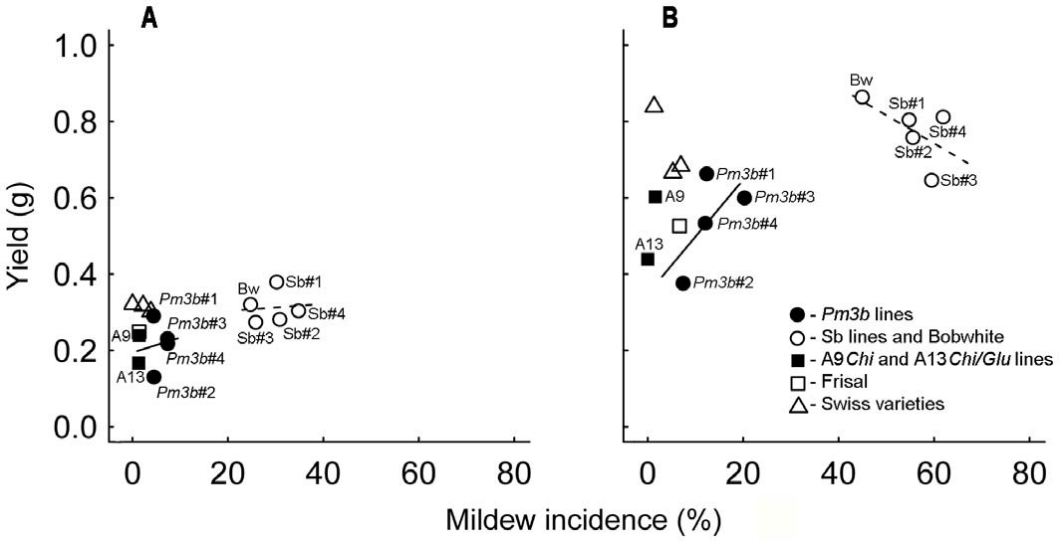
The relationship between mildew incidence and yield in 15 wheat lines. Left chart (A): soil with low nutrient level. Right column (B): soil with high nutrient level (fertilized subplots). The solid and dotted lines are linear regression lines for the groups of means for transgenic *Pm3b* lines and for control Sb lines and variety Bobwhite. Mildew incidence is a percentage of plants infected with the pathogen. The data for 15 wheat competitive environments are pooled.

In accordance with the different transformation events leading to the four *Pm3b* lines with different expression levels, we found significant differences between the four transgenic lines in their performance and interactions with the different competitive environments. Thus, line *Pm3b*#2, which showed the highest resistance to powdery mildew, was the weakest competitor, re-enforcing the view that transgene-caused, high mildew resistance was negatively correlated with plant performance. When the average yields are plotted against the average mildew incidence for all Frisal and Bobwhite lines (Figure 3) it can be seen that the relationship is positive at low infection levels for GM lines of Bobwhite and negative at high infection levels for control lines of Bobwhite (in fertilized subplots). This suggests that at very high levels of plant defense there is no gain for a plant to become even more resistant. Rather, increased resistance in this case could lead to a reduction in performance.

All *Pm3b* lines had enhanced transgene expression compared with the normal expression in the wheat landrace from which the *pm3b* gene originated (see [31], [32] for details). The line *Pm3b*#2, however, showed fivefold higher expression than the average of lines *Pm3b*#1, *Pm3b*#3 and *Pm3b*#4 [32]. This indicates that the overexpression of the gene that confers resistance could underlie the changes in the plants' interactions with their environment. Because the corresponding control lines passed through the same transformation procedure as *Pm3b* lines but did not show reduced competitive performance, we assume that the reduced performance in *Pm3b* lines was a consequence of the physiological costs they paid for the increased resistance to the pathogen [5], [6], [52]. Another GM line, *Pm3b*#3, had only minor or no performance differences compared with its control line. According to the gene expression and segregation analysis data [32], this line showed transgene silencing of different intensity in a large proportion of the plants and segregation in resistance (about 44% susceptible plants in the sixth generation). It also showed higher average mildew incidence than the other three GM lines in our phytometer experiment (Figure 1). The gene silencing could be an explanation for the lower costs of resistance found in *Pm3b*#3 line.

The data obtained at individual plant level in the phytometer experiment supported the results of our previous glasshouse and plot-level field assessments of the same *Pm3b* and Sb lines grown from seed [31], [32]. The transplanted phytometers of *Pm3b*#2 line showed the same altered phenotypes as did the sown plants [31], [32]. These alterations, strong resistance to mildew and weaker performance of the *Pm3b*#2 line under competition most likely were a consequence of the transgene overexpression and not due to events occurring during tissue culture because the sister plants of control line Sb#2 had undergone the same tissue culture events. In a previous study [31], where we assessed the ecological behaviour of sown plants of the different lines of Bobwhite at subplot level, we found that the transgenic lines *Pm3b*#1–4, compared with their sister lines, also had increased levels of ergot infection, suggesting that further, non-observed pleiotropic effects might have influenced the yield of GM plants (including phytometers) in our study.

Line A13 *Chi/Glu*, which expressed both *chitinase* and *glucanase* transgenes, had lower yield and seed number than the Frisal control line. In accordance with this observation, line A13 *Chi/Glu* also showed an increased resistance compared with the control line in fertilized subplots. Again it appears that additional investment into pathogen resistance, which was already elevated in the Frisal control line, was costly for the Frisal GM line expressing two transgenes. That performance was not reduced in the Frisal line expressing only one transgene (A9 *Chi*) suggests that the degree of defense matters for the costs of defense. We conclude that a high constitutive level of mildew resistance has negative effects on the performance of GM wheat plants and thus reduces their potential to persist in conventional agricultural fields. It could be that lower levels of intrinsic resistance to pathogens might produce better-performing GM plants. From a risk perspective, however, such plants would have to be evaluated again in a range of biotic and abiotic environments in similar experiments as the one presented here to test their or their offspring's potential to successfully compete with non-GM plants.

### Main Effects of Environments

Our second question was how variation in the abiotic (fertilization) and biotic environment (competition with other wheat lines) may influence resistance to mildew and the performance of phytometer plants. Nutrient addition enhanced powdery mildew incidence in both transgenic and conventional wheat lines. Similar effects were reported in previous studies with non-transgenic plants, where the severity of mildew infection was shown to be related to the nitrogen supply of the host [53]–[56]. Lines with high mildew incidence proved to be infective environments as shown by the higher mildew incidence of phytometers in these (Figure 1). This is a well-known epidemiological effect [57] and relevant when considering planting mixed-line crops because in the same way as more susceptible neighbors can increase infection in less susceptible target plants, so can more resistant neighbors reduce infection in less resistant target plants. In a further field experiment we found that indeed overall mildew incidence in line mixtures was lower than in the average single-line stand (S. Zeller, O. Kalinina & B. Schmid, unpublished data), an observation previously made in a genetic diversity experiment with the wild plant species *Solidago canadensis* [58].

Fertilization enhanced plant growth and reproduction in all the investigated wheat lines. In addition, the performance of the phytometer plants was strongly influenced by the type of competitive environment. Phytometers planted with transgenic *Pm3b* lines as competitors outperformed those planted into competitive environments of Sb lines. This is in accordance with the results of the analysis of the main effects of transgenes (see previous section). Phytometers which had Frisal variety as a competitive environment generally had weaker performance than those in Bobwhite environments. The congruence between phytometer-line and competitive environment-line effects, i.e. high-performing phytometer lines also providing highly competitive environments thus in turn reducing phytometer performance, indicates that phytometers do provide realistic measures of competitive ability.

### Overall Transgene × Environment Interactions

The third question asked whether transgenic wheat lines responded to variations in nutrient and competitive environments in the same way as did conventional lines. The difference in mildew incidence between GM lines and control increased with the addition of nutrients. A similar effect has been previously described in non-transgenic plants, where the increased severity of infection due to fertilization was more pronounced in susceptible than in resistant crop varieties and therefore the magnitude of the difference between these varieties increased with nutrient addition [55]. In accordance with these observations, the difference in mildew incidence between the transgenic lines and control lines also became stronger in mildew-susceptible than in mildew-resistant competitive environments.

Significant transgene × competitive environment interactions were observed for the majority of fitness-related traits and reflected more sensitive responses to competition for transgenic *Pm3b* lines of variety Bobwhite than for other lines. Our findings indicate that a single gene that confers constitutive resistance to a pathogen might strongly affect genotype × environment interactions if expressed at a high level, making ecological costs of resistance apparent even in the presence of the pathogen. The fact that the differences between GM and control lines in pathogen level and plant performance vary depending on the environment points to the importance of testing transgenic plants under a set of biotic and abiotic environments in realistic field conditions [16], [17]. The phytometer approach [21], [22] could be a useful tool for this kind of study.

Using this approach we could assess competitive interactions and the response to fertilizer treatments in 15 different transgenic and conventional wheat lines simultaneously on a relatively small area of less than 130 m^2^ in the field. An advantage of the phytometer approach is the possibility to incorporate several biotic and abiotic factors that might affect the performance and competitive ability of test plants simultaneously into a single and comprehensive experimental setting [21], [22], [25]. In addition to measuring the competitiveness of the individual phytometer plants, the experimental design also allowed us to assess the competitive strength of the environment provided by each wheat line. Where the phytometers benefited from being in a certain competitive environment it indicated that the line representing this environment was not a strong competitor. Furthermore, the overall effect that phytometers performed better in the neighborhood of plants from other lines (away) rather than their own line (home) suggests that line mixtures should perform better than the average line monoculture at plot level (see next section); a positive biodiversity effect normally tested with large setups of plots varying in diversity level [28]. In the future the phytometer approach could be used in field studies of transgenic plants to facilitate the identification of promising new breeds and increase the flexibility and power of ecological risk assessment.

### Relative Performance in Home vs. Away Environments

The fourth question was whether transgenic and non-transgenic lines behave differently if planted into their own (home) rather than into different lines as competitive environments (away). Most of the phytometer plants benefited if their neighbors belonged to a different line (mixture effect). This is consistent with findings in biodiversity experiments [28]. The transgenic *Pm3b* lines of variety Bobwhite, however, showed lower relative values (performance in “away” as compared to “home” environments) than the corresponding control lines for four out of six fitness-related traits. Only the line with partial gene silencing, *Pm3b*#3, showed no such costs under competition with the other lines. Because the GM line *Pm3b*#2 suffered most in mixtures, it appears that this line paid a particularly high fitness costs for its elevated mildew resistance under competition. This observation supports the recent findings that competition might increase the magnitude of the costs of resistance [10], [11], [59]. Our results, however, also point to the importance of the type of the competitor and the expression level of the resistance gene. The resistant line with the highest transgene expression, *Pm3b*#2, appeared to be especially sensitive to inter-line competition, whereas the differences between this line and its sister control line became smaller when the competitor was represented by its own genotype. Interestingly, the reduced performance and fecundity under competition with the other wheat lines (relative performance) was also observed in the *Pm3b*#4 line (Figure 2B) which is known to carry an additional non-functional truncated copy of the transgene. As the level of the gene expression did not differ strongly between the lines *Pm3b*#1 and *Pm3b*#4, it could be speculated that the impaired competitiveness of this line was caused by position effects via the disruption of endogenous genes [13], [32].

Transgenic plants with unintended phenotypes, including low fecundity, often arise during molecular plant breeding [60], [61]. They are usually detected early and their ecological performance is not further investigated [62]. In our case, however, the transgenic lines had higher performance than their non-transgenic control lines in the glasshouse under high pathogen pressure and only in the field this fitness advantage reversed [31]. Although there have been several studies that measured the costs of resistance in transgenic plants or in plants with induced defenses [3], [5], [6], [8]–[11], [59], [63], [64], only few of those have considered the effects of intra- and interline competition on the costs of resistance [11], [63], [64]. One of these studies found that the benefits of transgenic resistance to herbivores in rice disappeared when the plants were grown in competition with other genotypes instead of a pure stand [63].

Our results confirm these precedents and demonstrate that a transgene increasing plant resistance to a pathogen and constitutively expressed at a high level may reduce rather than increase a plant's competitive ability and thus lower its probability to persist outside its own field. An early study of Crawley *et al.* showed that herbicide-tolerant transgenic lines of rape showed no evidence to be more successful or more invasive than their conventional counterparts in the *absence* of herbicide treatment and even showed weaker invasive potential in some aspects, such as in seed survival after burial [65]. In our experiment, however, the costs for plant fitness and competitiveness could be observed even in the *presence* of the pathogen against which the GM lines had increased resistance. Very likely we would have observed even higher costs in our study if the pathogen would have been excluded in our field trial.

Apart from these findings, nutrient addition negatively affected the ability of plants to coexist in the mixtures. This supports the theory that fertilization increases competition between genotypes or species for scarce resources and in particular light [66], [67]. It would therefore be even more difficult for competitively weak transgenic plants to persist in well-fertilized agricultural habitats.

### Conclusions

In conclusion, this study shows that a single gene conferring resistance against a particular fungal pathogen can have large and negative effects on plant performance under realistic field conditions even if these conditions include the presence of the pathogen. We interpret these large costs in resistant plants as a consequence of altered gene regulation, in particular enhanced gene expression level, which was here achieved with a strong promoter introduced with the gene that confers resistance. This indicates that altered regulation in a single gene may strongly affect plant fitness and the way the plant interacts with the environment, in particular changing a plant's competitive ability.

## Supporting Information

Figure S1 Design of the phytometer experiment.
(PDF)

Figure S2 The structure of orthogonal contrasts used in the extended ANOVA models.
(PDF)

Table S1 Analysis of deviance table showing the effects of fertilizer, competitive environment, differences between GM and non-GM lines and their interactions on mildew incidence.
(PDF)

Table S2 ANOVA table showing the effects of fertilizer, competitive environment, differences between GM and non-GM lines and their interactions on three yield characteristics.
(PDF)

Table S3 ANOVA table showing the effects of fertilizer, competitive environment, differences between GM and non-GM lines and their interactions on phenological stage, plant height and vegetative mass.
(PDF)

Table S4 ANOVA table showing the effects of fertilizer, competitive environment, differences between GM and non-GM lines and their interactions on three relative yield characteristics.
(PDF)

Table S5 ANOVA table showing the effects of fertilizer, competitive environment, differences between GM and non-GM lines and their interactions on relative phenological stage, plant height and vegetative mass.
(PDF)
