# Supplementary material for: Competitive Performance of Transgenic Wheat Resistant to Powdery Mildew
Source: PLoS One. 2011 Nov 23;6(11):e28091. doi: 10.1371/journal.pone.0028091 (PMC3223217; doi:10.1371/journal.pone.0028091)
Supplement: Figure S1 — Design of the phytometer experiment. (PDF) [file pone.0028091.s001.pdf]

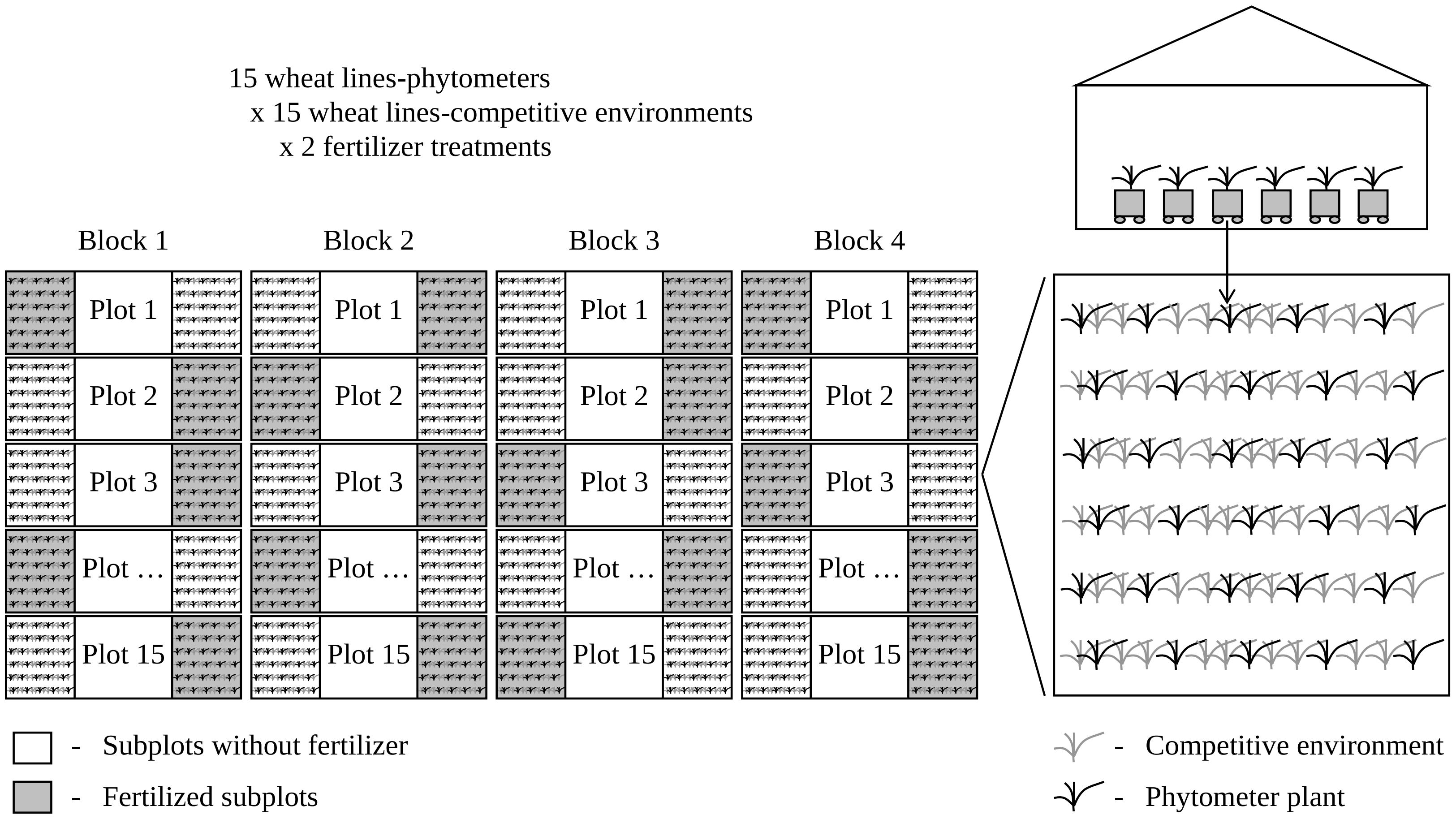

Figure S1. Design of the phytometer experiment.
