# Supplementary material for: Competitive Performance of Transgenic Wheat Resistant to Powdery Mildew
Source: PLoS One. 2011 Nov 23;6(11):e28091. doi: 10.1371/journal.pone.0028091 (PMC3223217; doi:10.1371/journal.pone.0028091)
Supplement: Figure S2 — The structure of orthogonal contrasts used in the extended ANOVA models. (PDF) [file pone.0028091.s002.pdf]

Version 1.

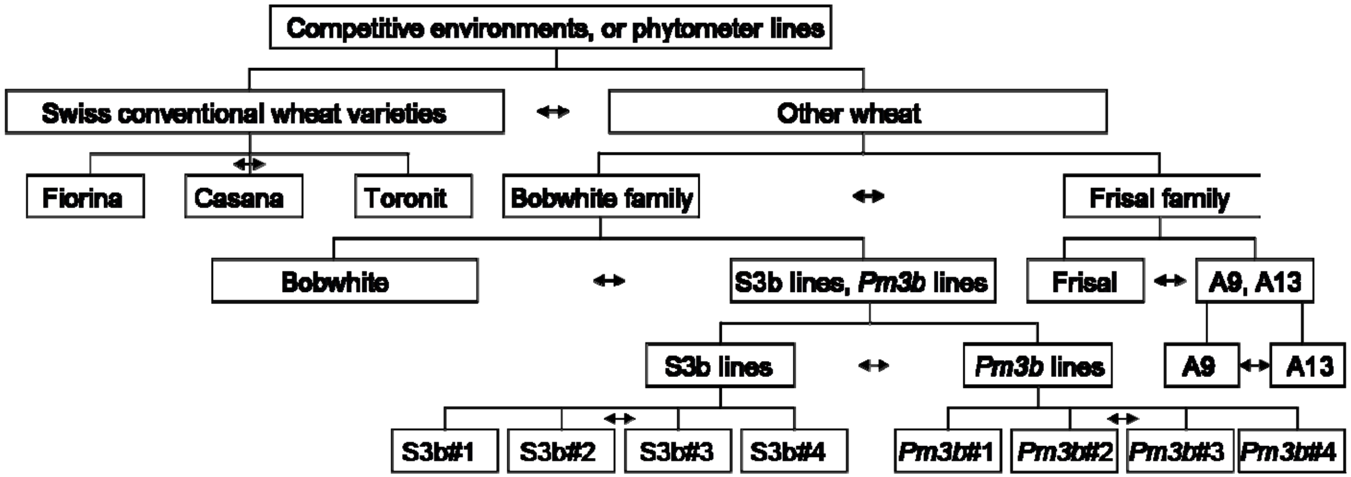

Version 2.

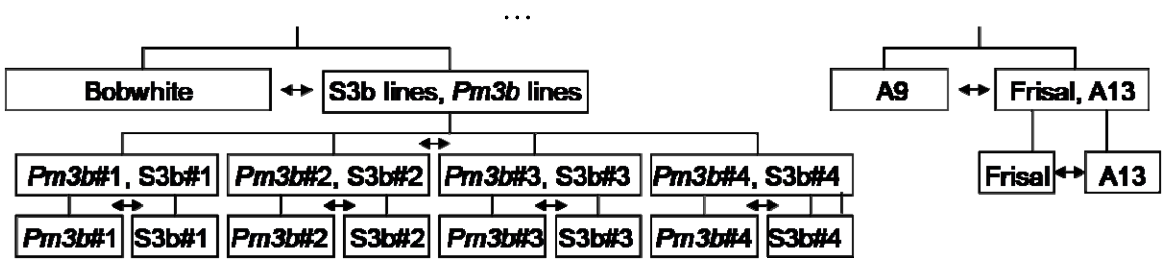

Version 3.

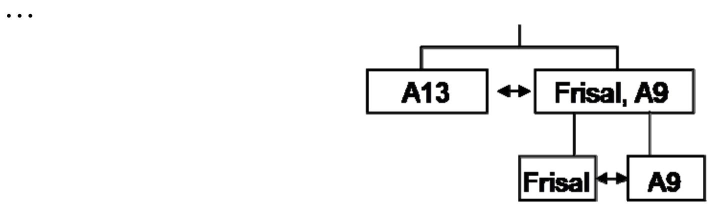

Figure S2. The structure of orthogonal contrasts used in the extended ANOVA models.
