# Supplementary material for: Competitive Performance of Transgenic Wheat Resistant to Powdery Mildew
Source: PLoS One. 2011 Nov 23;6(11):e28091. doi: 10.1371/journal.pone.0028091 (PMC3223217; doi:10.1371/journal.pone.0028091)
Supplement: Table S1 — Analysis of deviance table showing the effects of fertilizer, competitive environment, differences between GM and non-GM lines and their interactions on mildew incidence. (PDF) [file pone.0028091.s003.pdf]

Table S1. Analysis of deviance table showing the effects of fertilizer, competitive environment, differences between GM and non-GM lines and their interactions on mildew incidence

Simple model

Source of variation	Mildew incidence		
	df	%SS	F pr.
Block	3	1.1	0.018
Competitive environment (Comp.env.)	14	5.2	0.001
Plot	42	4.3	0.040
Fertilizer	1	3.1	<.001
Comp.env.×Fertilizer	14	1.2	0.202
Subplot	45	2.7	0.949
Phytometer lines	14	26.8	<.001
Comp.env.×Phytometer lines	196	7.1	0.067
Plot×Phytometer lines	593	18.2	<.001
Phytometer lines×Fertilizer	14	1.2	<.001
Residual	1513	29.0	
Total	2449	100	

Extended model

Source of variation	Mildew incidence		
	df	%SS	F pr.
Block	3	1.1	0.018
Competitive environment (Comp.env.)	14	5.2	0.001
Plot	42	4.3	0.040
Fertilizer	1	3.1	<.001
Comp.env.×Fertilizer	14	1.2	0.202
Subplot	45	2.7	<.001
Phytometer contrasts (Phytometer lines effect):			
Swiss vs. other wheat	1	5.7	<.001
3 conventional Swiss varieties	2	0.3	<.001
Bobwhite vs. Frisal	1	7.6	<.001
Bobwhite vs. Sb lines	1	0.3	<.001
Pm3b lines vs. Sb lines	1	12.4	<.001
4 Sb lines	3	0.1	0.059
4 *Pm3b* lines	3	0.3	0.001
A9 *Chi* and A13 *Chi/Glu* vs. Frisal	1	0.1	0.008
A9 *Chi* vs. A13 *Chi/Glu*	1	0.0	0.331
Pairwise comparisons:			
Pm3b#1 vs. Sb#1	1	3.1	<.001
Pm3b#2 vs. Sb#2	1	3.7	<.001
Pm3b#3 vs. Sb#3	1	1.7	<.001
Pm3b#4 vs. Sb#4	1	4.1	<.001
A9 *Chi* vs. Frisal	1	0.1	0.0073
A13 *Chi/Glu* vs. Frisal	1	0.1	0.006
Comp.env.×Swiss vs. other wheat	14	0.4	0.069
Comp.env.×3 conventional Swiss varieties	28	0.7	0.671
Comp.env.×Bobwhite vs. Frisal	14	0.7	0.088
Comp.env.×Bobwhite vs. Sb lines	14	0.3	0.728
Comp.env.× *Pm3b* lines vs. Sb lines	14	0.8	0.024
Comp.env.×4 Sb lines	42	2.0	0.014
Comp.env.×4 *Pm3b* lines	42	1.6	0.138
Comp.env.×A9 *Chi* and A13 *Chi/Glu* vs. Frisal	14	0.3	0.756
Comp.env.×A9 *Chi* vs. A13 *Chi/Glu*	14	0.2	0.947
Plot×Phytometer lines	593	18.2	<.001
Fertilizer×Swiss vs. other wheat	1	0.1	0.119
Fertilizer×3 conventional Swiss varieties	2	0.0	0.487
Fertilizer×Bobwhite vs. Frisal	1	0.0	0.758
Fertilizer×Bobwhite vs. Sb lines	1	0.0	0.843
Fertilizer×*Pm3b* lines vs. Sb lines	1	0.2	<.001
Fertilizer×4 Sb lines	3	0.3	0.002
Fertilizer×4 *Pm3b* lines	3	0.1	0.225
Fertilizer×A9 *Chi* and A13 *Chi/Glu* vs. Frisal	1	0.2	0.002
Fertilizer×A9 *Chi* vs. A13 *Chi/Glu*	1	0.4	<.001
Residual	1513	29.0	
Total	2449	100	
