# Supplementary material for: Competitive Performance of Transgenic Wheat Resistant to Powdery Mildew
Source: PLoS One. 2011 Nov 23;6(11):e28091. doi: 10.1371/journal.pone.0028091 (PMC3223217; doi:10.1371/journal.pone.0028091)
Supplement: Table S2 — ANOVA table showing the effects of fertilizer, competitive environment, differences between GM and non-GM lines and their interactions on three yield characteristics. (PDF) [file pone.0028091.s004.pdf]

Table S2. ANOVA table showing the effects of fertilizer, competitive environment, differences between GM and non-GM lines and their interactions on three yield characteristics

Simple model

Source of variation	Yield (log)			Spike number (log)			Seed number (log)		
	df	%SS	F pr.	df	%SS	F pr.	df	%SS	F pr.
Block	3	4.2	<.001	3	2.9	<.001	3	3.5	<.001
Competitive environment	14	8.3	<.001	14	5.9	<.001	14	8.5	<.001
Plot	42	6.4	<.001	42	3.4	0.376	42	5.5	<.001
Fertilizer	1	17.2	<.001	1	9.7	<.001	1	17.0	<.001
Comp.env.×Fertilizer	14	0.5	0.596	14	0.8	0.709	14	0.4	0.696
Subplot	45	1.9	0.007	45	3.3	<.001	45	1.8	0.017
Phytometer lines	14	5.9	<.001	14	2.0	<.001	14	6.7	<.001
Comp.env.×Phytometer lines	196	5.4	0.071	196	6.1	0.583	196	5.7	0.058
Plot×Phytometer lines	593	13.9	0.888	598	19.2	0.171	593	14.3	0.818
Phytometer lines×Fertilizer	14	0.2	0.896	14	0.7	0.085	14	0.2	0.811
Residual	1406	36.0		1522	45.9		1406	36.2	
Total	2342	100		2463	100		2342	100	

Extended model

Source of variation	Yield (log)			Spike number (log)			Seed number (log)		
	df	%SS	F pr.	df	%SS	F pr.	df	%SS	F pr.
Block	3	4.2	<.001	3	2.9	<.001	3	3.5	<.001
Competitive environment contrasts:									
Swiss vs. other wheat	1	1.7	0.002	1	0.7	0.006	1	1.8	0.171
3 conventional Swiss varieties	2	0.5	0.183	2	0.9	0.009	2	0.6	0.123
Bobwhite vs. Frisal	1	0.4	0.137	1	0.0	0.679	1	0.4	0.072
Bobwhite vs. Sb lines	1	0.0	0.959	1	0.1	0.182	1	0.0	0.840
Pm3b lines vs. Sb lines	1	2.7	<.001	1	2.4	<.001	1	3.1	<.001
4 Sb lines	3	1.3	0.044	3	0.1	0.724	3	1.2	0.035
4 *Pm3b* lines	3	1.3	0.047	3	1.7	0.001	3	1.2	0.038
A9 *Chi* and A13 *Chi/Glu* vs. Frisal	1	0.0	0.814	1	0.0	0.909	1	0.0	0.960
A9 *Chi* vs. A13 *Chi/Glu*	1	0.3	0.149	1	0.0	0.841	1	0.2	0.241
Plot	42	6.4	<.001	42	3.4	0.376	42	5.5	<.001
Fertilizer	1	17.2	<.001	1	9.7	<.001	1	17.0	<.001
Comp.env.×Fertilizer	14	0.5	0.596	14	0.8	0.709	14	0.4	0.696
Subplot	45	1.8	0.007	45	3.3	<.001	45	1.8	0.017
Phytometer contrasts:									
Swiss vs. other wheat	1	0.9	<.001	1	0.1	0.073	1	0.2	0.015
3 conventional Swiss varieties	2	0.1	0.204	2	0.0	0.658	2	0.1	0.068
Bobwhite vs. Frisal	1	0.3	<.001	1	1.1	<.001	1	0.6	<.001
Bobwhite vs. Sb lines	1	0.6	<.001	1	0.0	0.459	1	1.2	<.001
Pm3b lines vs. Sb lines	1	1.8	<.001	1	0.0	0.609	1	2.8	<.001
4 Sb lines	3	0.1	0.129	3	0.1	0.372	3	0.1	0.158
4 *Pm3b* lines	3	1.8	<.001	3	0.7	<.001	3	1.2	<.001
A9 *Chi* and A13 *Chi/Glu* vs. Frisal	1	0.0	0.441	1	0.0	0.647	1	0.1	0.053
A9 *Chi* vs. A13 *Chi/Glu*	1	0.3	<.001	1	0.0	0.759	1	0.4	<.001
Pairwise comparisons:									
Pm3b#1 vs. Sb#1	1	0.1	0.047	1	0.0	0.759	1	0.2	0.005
Pm3b#2 vs. Sb#2	1	2.1	<.001	1	0.1	0.095	1	1.8	<.001
Pm3b#3 vs. Sb#3	1	0.1	0.067	1	0.0	0.426	1	0.3	0.001
Pm3b#4 vs. Sb#4	1	0.4	<.001	1	0.1	0.104	1	1.1	<.001
A9 *Chi* vs. Frisal	1	0.0	0.293	1	0.0	0.582	1	0.0	0.698
A13 *Chi/Glu* vs. Frisal	1	0.2	0.017	1	0.0	0.795	1	0.4	<.001
Comp.env.×Phytometer lines	196	5.4	0.071	196	6.1	0.583	196	5.7	0.058
Plot×Phytometer lines	593	13.9	0.888	598	19.2	0.171	593	14.3	0.818
Fertilizer×Swiss vs. other wheat	1	0.0	0.946	1	0.0	0.287	1	0.0	0.534
Fertilizer×3 conventional Swiss varieties	2	0.0	0.964	2	0.1	0.438	2	0.0	0.798
Fertilizer×Bobwhite vs. Frisal	1	0.1	0.107	1	0.1	0.062	1	0.0	0.17
Fertilizer×Bobwhite vs. Sb lines	1	0.0	0.781	1	0.0	0.236	1	0.0	0.787
Fertilizer×*Pm3b* lines vs. Sb lines	1	0.0	0.415	1	0.0	0.503	1	0.0	0.642
Fertilizer×4 Sb lines	3	0.0	0.923	3	0.2	0.096	3	0.0	0.873
Fertilizer×4 *Pm3b* lines	3	0.1	0.486	3	0.1	0.613	3	0.1	0.369
Fertilizer×A9 *Chi* and A13 *Chi/Glu* vs. Frisal	1	0.0	0.253	1	0.1	0.128	1	0.1	0.152
Fertilizer×A9 *Chi* vs. A13 *Chi/Glu*	1	0.0	0.642	1	0.1	0.078	1	0.0	0.54
Residual	1406	36.0		1522	45.9		1406	36.2	
Total	2342	100		2463	100		2342	100.0	
