# Supplementary material for: Competitive Performance of Transgenic Wheat Resistant to Powdery Mildew
Source: PLoS One. 2011 Nov 23;6(11):e28091. doi: 10.1371/journal.pone.0028091 (PMC3223217; doi:10.1371/journal.pone.0028091)
Supplement: Table S3 — ANOVA table showing the effects of fertilizer, competitive environment, differences between GM and non-GM lines and their interactions on phenological stage, plant height and vegetative mass. (PDF) [file pone.0028091.s005.pdf]

Table S3. ANOVA table showing the effects of fertilizer, competitive environment, differences between GM and non-GM lines and their interactions on phenological stage, plant height and vegetative mass

Simple model

Source of variation	Vegetative mass (log)			Plant height (log)			Phenological stage (log)		
	df	%SS	F pr.	df	%SS	F pr.	df	%SS	F pr.
Block	3	2.2	0.005	3	31.8	<.001	3	28.1	<.001
Competitive environment	14	10.1	<.001	14	0.6	0.537	14	1.0	0.136
Plot	42	6.5	<.001	42	2.1	0.030	42	2.0	0.203
Fertilizer	1	27.7	<.001	1	7.0	<.001	1	5.1	<.001
Comp.env.×Fertilizer	14	0.4	0.904	14	0.9	0.017	14	0.4	0.674
Subplot	45	2.2	<.001	45	1.3	<.001	45	1.6	<.001
Phytometer lines	14	5.8	<.001	14	24.2	<.001	14	20.4	<.001
Comp.env.×Phytometer lines	196	3.6	0.896	196	2.0	0.846	196	2.4	0.999
Plot×Phytometer lines	597	12.7	0.082	630	7.4	<.001	630	11.8	<.001
Phytometer lines×Fertilizer	14	0.4	0.160	14	0.6	<.001	14	0.4	0.001
Residual	1473	28.5		2625	22.2		2594	26.9	
Total	2413	100		3598	100		3567	100	

Extended model

Source of variation	Vegetative mass (log)			Plant height (log)			Phenological stage (log)		
	df	%SS	F pr.	df	%SS	F pr.	df	%SS	F pr.
Block	3	2.2	0.005	3	31.8	<.001	3	28.1	<.001
Competitive environment contrasts:									
Swiss vs. other wheat	1	2.4	<.001	1	0.1	0.210	1	0.0	0.691
3 conventional Swiss varieties	2	1.0	0.050	2	0.1	0.489	2	0.2	0.089
Bobwhite vs. Frisal	1	0.3	0.175	1	0.1	0.200	1	0.4	0.004
Bobwhite vs. Sb lines	1	0.1	0.374	1	0.0	0.604	1	0.0	0.35
Pm3b lines vs. Sb lines	1	2.6	<.001	1	0.0	0.969	1	0.0	0.692
4 Sb lines	3	1.7	0.018	3	0.1	0.652	3	0.0	0.998
4 *Pm3b* lines	3	1.7	0.017	3	0.1	0.402	3	0.0	0.878
A9 *Chi* and A13 *Chi/Glu* vs. Frisal	1	0.0	0.703	1	0.1	0.115	1	0.3	0.025
A9 *Chi* vs. A13 *Chi/Glu*	1	0.2	0.297	1	0.0	0.399	1	0.0	0.921
Plot	42	6.4	<.001	42	2.1	0.030	42	2.0	0.203
Fertilizer	1	27.7	<.001	1	7.0	<.001	1	5.1	<.001
Comp.env.×Fertilizer	14	0.4	0.904	14	0.9	0.017	14	0.4	0.674
Subplot	45	2.2	<.001	45	1.3	<.001	45	1.6	<.001
Phytometer contrasts:									
Swiss vs. other wheat	1	0.0	0.395	1	6.0	<.001	1	3.3	<.001
3 conventional Swiss varieties	2	0.1	0.169	2	0.4	<.001	2	0.4	<.001
Bobwhite vs. Frisal	1	2.8	<.001	1	17.1	<.001	1	16.2	<.001
Bobwhite vs. Sb lines	1	0.1	0.013	1	0.0	0.224	1	0.0	0.111
Pm3b lines vs. Sb lines	1	0.2	<.001	1	0.2	<.001	1	0.2	<.001
4 Sb lines	3	0.3	<.001	3	0.0	0.515	3	0.0	0.591
4 *Pm3b* lines	3	2.2	<.001	3	0.4	<.001	3	0.1	0.023
A9 *Chi* and A13 *Chi/Glu* vs. Frisal	1	0.0	0.381	1	0.0	0.241	1	0.0	0.508
A9 *Chi* vs. A13 *Chi/Glu*	1	0.0	0.245	1	0.0	0.088	1	0.0	0.195
Pairwise comparisons:									
Pm3b#1 vs. Sb#1	1	0.0	0.015	1	0.0	0.599	1	0.0	0.187
Pm3b#2 vs. Sb#2	1	0.0	<.001	1	0.4	<.001	1	0.2	<.001
Pm3b#3 vs. Sb#3	1	0.0	0.768	1	0.0	0.060	1	0.0	0.139
Pm3b#4 vs. Sb#4	1	0.0	0.113	1	0.0	0.273	1	0.1	0.008
A9 *Chi* vs. Frisal	1	0.0	0.872	1	0.0	0.871	1	0.0	0.223
A13 *Chi/Glu* vs. Frisal	1	0.0	0.185	1	0.0	0.062	1	0.0	0.945
Comp.env.×Phytometer lines	196	3.6	0.896	196	2.0	0.846	196	2.4	0.999
Plot×Phytometer lines	597	12.7	0.082	630	7.4	<.001	630	11.8	<.001
Fertilizer×Swiss vs. other wheat	1	0.1	0.114	1	0.1	<.001	1	0.0	0.892
Fertilizer×3 conventional Swiss varieties	2	0.2	0.019	2	0.0	0.603	2	0.1	0.009
Fertilizer×Bobwhite vs. Frisal	1	0.1	0.095	1	0.3	<.001	1	0.1	0.012
Fertilizer×Bobwhite vs. Sb lines	1	0.0	0.694	1	0.0	0.842	1	0.0	0.196
Fertilizer×*Pm3b* lines vs. Sb lines	1	0.0	0.834	1	0.0	0.830	1	0.0	0.468
Fertilizer×4 Sb lines	3	0.0	0.820	3	0.1	0.024	3	0.0	0.443
Fertilizer×4 *Pm3b* lines	3	0.1	0.359	3	0.1	0.061	3	0.1	0.132
Fertilizer×A9 *Chi* and A13 *Chi/Glu* vs. Frisal	1	0.0	0.426	1	0.0	0.022	1	0.0	0.214
Fertilizer×A9 *Chi* vs. A13 *Chi/Glu*	1	0.0	0.319	1	0.0	0.503	1	0.1	0.004
Residual	1473	28.5		2625	22.2		2594	26.9	
Total	2413	100		3598	100		3567	100	
