# Supplementary material for: Competitive Performance of Transgenic Wheat Resistant to Powdery Mildew
Source: PLoS One. 2011 Nov 23;6(11):e28091. doi: 10.1371/journal.pone.0028091 (PMC3223217; doi:10.1371/journal.pone.0028091)
Supplement: Table S4 — ANOVA table showing the effects of fertilizer, competitive environment, differences between GM and non-GM lines and their interactions on three relative yield characteristics. (PDF) [file pone.0028091.s006.pdf]

Table S4. ANOVA table showing the effects of fertilizer, competitive environment, differences between GM and non-GM lines and their interactions on three relative yield characteristics

Simple model

Source of variation	Yield (log)			Spike number (log)			Seed number (log)		
	df	%SS	F pr.	df	%SS	F pr.	df	%SS	F pr.
Overall mean	1	0.4	0.009	1	0.0	0.503	1	0.2	0.032
Block	3	1.4	0.021	3	1.9	0.001	3	1.8	0.002
Competitive environment	14	7.5	<.001	14	5.9	<.001	14	8.9	<.001
Plot	47	5.9	0.075	47	4.3	0.287	43	4.6	0.030
Fertilizer	1	0.5	0.014	1	0.1	0.290	1	2.3	<.001
Comp.env.×Fertilizer	14	0.6	0.917	14	0.3	0.991	14	0.3	0.977
Subplot	43	3.5	0.059	44	3.4	0.007	46	2.8	0.072
Phytometer lines	14	12.7	<.001	14	13.5	<.001	14	9.9	<.001
Comp.env.×Phytometer lines	180	6.7	0.999	180	6.8	0.999	182	6.8	0.997
Plot×Phytometer lines	502	35.6	0.029	520	40.6	<.001	552	29.2	0.031
Phytometer lines×Fertilizer	13	3.9	<.001	14	3.4	<.001	14	8.7	<.001
Residual	363	21.3		421	19.8		550	24.8	
Total	1195	100		1273	100		1433	100.0	

Extended model

Source of variation	Yield (log)			Spike number (log)			Seed number (log)		
	df	%SS	F pr.	df	%SS	F pr.	df	%SS	F pr.
Overall mean	1	0.4	0.009	1	0.0	0.503	1	1.8	0.002
Block	3	1.4	0.021	3	1.9	0.001	3	8.9	<.001
Competitive environment	14	7.5	<.001	14	5.9	<.001	14	4.6	0.030
Plot	47	5.9	0.075	47	4.3	0.287	43	2.3	<.001
Fertilizer	1	0.5	0.014	1	0.1	0.290	1	0.3	0.977
Comp.env.×Fertilizer	14	0.6	0.917	14	0.3	0.991	14	2.8	0.988
Subplot	43	3.5	0.059	44	3.4	0.007	46	1.8	0.002
Phytometer contrasts:									
Swiss vs. other wheat	1	1.1	<.001	1	0.2	0.022	1	0.7	<.001
3 conventional Swiss varieties	2	0.2	0.219	2	1.3	<.001	2	0.2	0.064
Bobwhite vs. Frisal	1	0.0	0.447	1	0.1	0.084	1	0.2	0.040
Bobwhite vs. Sb lines	1	0.8	<.001	1	1.2	<.001	1	0.1	0.116
Pm3b lines vs. Sb lines	1	5.7	<.001	1	4.4	<.001	1	4.4	<.001
4 Sb lines	3	1.3	<.001	3	1.5	<.001	3	1.1	<.001
4 *Pm3b* lines	3	3.5	<.001	3	4.7	<.001	3	2.9	<.001
A9 *Chi* and A13 *Chi/Glu* vs. Frisal	1	0.0	0.543	1	0.0	0.396	1	0.0	0.721
A9 *Chi* vs. A13 *Chi/Glu*	1	0.1	0.240	1	0.0	0.533	1	0.3	0.013
Pairwise comparisons:									
Pm3b#1 vs. Sb#1	1	1.5	<.001	1	0.3	0.010	1	0.6	<.001
Pm3b#2 vs. Sb#2	1	4.3	<.001	1	4.3	<.001	1	5.2	<.001
Pm3b#3 vs. Sb#3	1	0.2	0.066	1	0.1	0.156	1	0.2	0.065
Pm3b#4 vs. Sb#4	1	0.6	0.002	1	3.7	<.001	1	0.5	0.001
A9 *Chi* vs. Frisal	1	0.0	0.561	1	0.1	0.125	1	0.1	0.120
A13 *Chi/Glu* vs. Frisal	1	0.0	0.541	1	0.0	0.373	1	0.0	0.357
Comp.env.×Phytometer lines	180	6.7	0.999	180	6.8	0.999	182	6.8	0.997
Plot×Phytometer lines	502	35.6	0.029	520	40.6	<.001	552	29.2	0.031
Fertilizer×Swiss vs. other wheat	1	0.1	0.188	1	0.1	0.100	1	0.3	0.016
Fertilizer×3 conventional Swiss varieties	2	0.6	0.009	2	0.2	0.091	2	1.3	<.001
Fertilizer×Bobwhite vs. Frisal	1	0.1	0.257	1	0.0	0.340	1	0.0	0.568
Fertilizer×Bobwhite vs. Sb lines	1	0.6	0.001	1	0.0	0.647	1	0.8	<.001
Fertilizer×*Pm3b* lines vs. Sb lines	1	1.3	<.001	1	1.0	<.001	1	2.1	<.001
Fertilizer×4 Sb lines	2	0.6	0.005	3	0.7	0.002	3	3.1	<.001
Fertilizer×4 *Pm3b* lines	3	0.5	0.033	3	0.5	0.010	3	0.4	0.045
Fertilizer×A9 *Chi* and A13 *Chi/Glu* vs. Frisal	1	0.0	0.731	1	0.0	0.828	1	0.2	0.029
Fertilizer×A9 *Chi* vs. A13 *Chi/Glu*	1	0.0	0.803	1	0.7	<.001	1	0.6	<.001
Residual	363	21.3		421	19.8		550	24.8	
Total	1195	100		1273	100		1433	100.0	
