# Supplementary material for: Competitive Performance of Transgenic Wheat Resistant to Powdery Mildew
Source: PLoS One. 2011 Nov 23;6(11):e28091. doi: 10.1371/journal.pone.0028091 (PMC3223217; doi:10.1371/journal.pone.0028091)
Supplement: Table S5 — ANOVA table showing the effects of fertilizer, competitive environment, differences between GM and non-GM lines and their interactions on relative phenological stage, plant height and vegetative mass. (PDF) [file pone.0028091.s007.pdf]

Table S5. ANOVA table showing the effects of fertilizer, competitive environment, differences between GM and non-GM lines and their interactions on relative phenological stage, plant height and vegetative mass

Simple model

Source of variation	Vegetative mass (log)			Plant height (log)			Phenological stage (log)		
	df	%SS	F pr.	df	%SS	F pr.	df	%SS	F pr.
Overall mean	1	0.6	<.001	1	0.2	0.027	1	0.5	0.001
Block	3	0.4	0.439	3	1.0	0.045	3	0.2	0.619
Competitive environment	14	9.5	<.001	14	2.1	0.217	14	2.2	0.259
Plot	47	7.2	0.013	47	5.2	0.723	47	5.9	0.005
Fertilizer	1	0.6	0.007	1	0.0	0.730	1	0.3	0.021
Comp.env.×Fertilizer	14	0.3	0.994	14	1.2	0.823	14	1.4	0.085
Subplot	44	3.4	0.015	44	5.8	<.001	44	2.5	0.144
Phytometer lines	14	13.5	<.001	14	7.7	<.001	14	10.2	<.001
Comp.env.×Phytometer lines	180	5.2	0.999	180	5.5	0.999	180	4.9	0.999
Plot×Phytometer lines	515	35.9	<.001	610	33.3	0.025	610	32.4	0.040
Phytometer lines×Fertilizer	14	3.3	<.001	14	3.5	<.001	14	5.6	<.001
Residual	403	20.1		735	34.5		731	33.9	
Total	1250	100		1677	100		1673	100	

Extended model

Source of variation	Vegetative mass (log)			Plant height (log)			Phenological stage (log)		
	df	%SS	F pr.	df	%SS	F pr.	df	%SS	F pr.
Overall mean	1	0.6	<.001	1	0.2	0.027	1	0.5	0.001
Block	3	0.4	0.439	3	1.0	0.045	3	0.2	0.619
Competitive environment	14	9.5	<.001	14	2.1	0.217	14	2.2	0.259
Plot	47	7.2	0.013	47	5.2	0.723	47	5.9	0.005
Fertilizer	1	0.6	0.007	1	0.0	0.730	1	0.3	0.021
Comp.env.×Fertilizer	14	0.3	0.994	14	1.2	0.823	14	1.4	0.085
Subplot	44	3.4	0.015	44	5.8	<.001	44	2.5	0.144
Phytometer contrasts:									
Swiss vs. other wheat	1	0.7	<.001	1	0.0	0.336	1	1.6	<.001
3 conventional Swiss varieties	2	1.4	<.001	2	2.1	<.001	2	1.5	<.001
Bobwhite vs. Frisal	1	0.7	<.001	1	0.0	0.617	1	0.0	0.685
Bobwhite vs. Sb lines	1	1.2	<.001	1	0.2	0.066	1	1.3	<.001
Pm3b lines vs. Sb lines	1	3.3	<.001	1	0.4	0.005	1	0.3	0.012
4 Sb lines	3	0.5	0.026	3	2.9	<.001	3	3.0	<.001
4 *Pm3b* lines	3	5.4	<.001	3	1.4	<.001	3	0.2	0.295
A9 *Chi* and A13 *Chi/Glu* vs. Frisal	1	0.0	0.325	1	0.6	<.001	1	2.0	<.001
A9 *Chi* vs. A13 *Chi/Glu*	1	0.2	0.026	1	0.0	0.832	1	0.3	0.013
Pairwise comparisons:									
Pm3b#1 vs. Sb#1	1	0.4	0.006	1	0.1	0.210	1	0.9	<.001
Pm3b#2 vs. Sb#2	1	5.0	<.001	1	0.4	0.003	1	0.8	<.001
Pm3b#3 vs. Sb#3	1	0.0	0.523	1	0.6	<.001	1	0.3	0.009
Pm3b#4 vs. Sb#4	1	0.6	0.001	1	0.2	0.025	1	0.4	0.004
A9 *Chi* vs. Frisal	1	0.3	0.025	1	0.3	0.017	1	2.4	<.001
A13 *Chi/Glu* vs. Frisal	1	0.0	0.862	1	0.2	0.029	1	1.1	<.001
Comp.env.×Phytometer lines	180	5.2	0.999	180	5.5	0.999	180	4.9	0.999
Plot×Phytometer lines	515	35.9	<.001	610	33.3	0.025	610	32.4	0.040
Fertilizer×Swiss vs. other wheat	1	0.1	0.226	1	0.0	0.781	1	0.2	0.030
Fertilizer×3 conventional Swiss varieties	2	0.0	0.907	2	0.6	0.001	2	0.1	0.373
Fertilizer×Bobwhite vs. Frisal	1	0.0	0.716	1	0.3	0.013	1	0.1	0.235
Fertilizer×Bobwhite vs. Sb lines	1	0.8	<.001	1	0.0	0.544	1	0.6	<.001
Fertilizer×*Pm3b* lines vs. Sb lines	1	1.2	<.001	1	0.6	<.001	1	1.0	<.001
Fertilizer×4 Sb lines	3	1.0	<.001	3	0.1	0.631	3	0.4	0.025
Fertilizer×4 *Pm3b* lines	3	0.1	0.611	3	0.8	0.001	3	0.7	0.002
Fertilizer×A9 *Chi* and A13 *Chi/Glu* vs. Frisal	1	0.1	0.199	1	0.0	0.468	1	0.0	0.320
Fertilizer×A9 *Chi* vs. A13 *Chi/Glu*	1	0.0	0.324	1	1.1	<.001	1	2.4	<.001
Residual	403	20.1		735	34.5		731	33.9	
Total	1250	100		1677	100		1673	100	
